# Artificial Intelligence and Advanced Technology in Glaucoma: A Review

**DOI:** 10.3390/jpm14101062

**Published:** 2024-10-16

**Authors:** Emanuele Tonti, Sofia Tonti, Flavia Mancini, Chiara Bonini, Leopoldo Spadea, Fabiana D’Esposito, Caterina Gagliano, Mutali Musa, Marco Zeppieri

**Affiliations:** 1 UOC Ophthalmology, Sant’Eugenio Hospital, 00144 Rome, Italy; emanuele.tonti@aslroma2.it; 2 Biomedical Engineering, Politecnico di Torino, 10129 Turin, Italy; 3 Eye Clinic, Policlinico Umberto I University Hospital, 00142 Rome, Italy; 4 Imperial College Ophthalmic Research Group (ICORG) Unit, Imperial College, 153-173 Marylebone Rd, London NW15QH, UK; 5 Department of Neurosciences, Reproductive Sciences and Dentistry, University of Naples Federico II, Via Pansini 5, 80131 Napoli, Italy; 6 Department of Medicine and Surgery, University of Enna “Kore”, Piazza dell’Università, 94100 Enna, Italy; 7 “G.B. Morgagni” Mediterranean Foundation, 95125 Catania, Italy; 8 Department of Optometry, University of Benin, Benin 300238, Nigeria; 9 Department of Ophthalmology, University Hospital of Udine, 33100 Udine, Italy

**Keywords:** glaucoma, ocular hypertension, artificial intelligence, personalized medicine, machine learning, deep learning

## Abstract

Background: Glaucoma is a leading cause of irreversible blindness worldwide, necessitating precise management strategies tailored to individual patient characteristics. Artificial intelligence (AI) holds promise in revolutionizing the approach to glaucoma care by providing personalized interventions. Aim: This review explores the current landscape of AI applications in the personalized management of glaucoma patients, highlighting advancements, challenges, and future directions. Methods: A systematic search of electronic databases, including PubMed, Scopus, and Web of Science, was conducted to identify relevant studies published up to 2024. Studies exploring the use of AI techniques in personalized management strategies for glaucoma patients were included. Results: The review identified diverse AI applications in glaucoma management, ranging from early detection and diagnosis to treatment optimization and prognosis prediction. Machine learning algorithms, particularly deep learning models, demonstrated high accuracy in diagnosing glaucoma from various imaging modalities such as optical coherence tomography (OCT) and visual field tests. AI-driven risk stratification tools facilitated personalized treatment decisions by integrating patient-specific data with predictive analytics, enhancing therapeutic outcomes while minimizing adverse effects. Moreover, AI-based teleophthalmology platforms enabled remote monitoring and timely intervention, improving patient access to specialized care. Conclusions: Integrating AI technologies in the personalized management of glaucoma patients holds immense potential for optimizing clinical decision-making, enhancing treatment efficacy, and mitigating disease progression. However, challenges such as data heterogeneity, model interpretability, and regulatory concerns warrant further investigation. Future research should focus on refining AI algorithms, validating their clinical utility through large-scale prospective studies, and ensuring seamless integration into routine clinical practice to realize the full benefits of personalized glaucoma care.

## 1. Introduction

Artificial intelligence (AI) has started completely transforming ophthalmology in recent years, significantly improving the identification and treatment of a wide range of eye disorders. The application of AI, especially deep learning methods, has demonstrated considerable potential in analyzing fundus imaging, optical coherence tomography (OCT), and visual field (VF) test data. These sophisticated algorithms provide increased accuracy and speed in identifying and diagnosing ocular diseases, ranging from corneal and choroidal problems to retinal and macular disorders [1].

AI in ophthalmology has a bright future ahead of it. Current research aims to improve these instruments further, augmenting their capacity to identify diseases in their early stages, predict the course of diseases, and support medical judgment. This review furthermore aims to provide an overview of the current state of the art regarding the use of AI in glaucoma. These developments potentially represent a huge step forward in ophthalmic treatment by improving clinical results and patient quality of life.

## 2. Materials and Methods

In order to give a thorough evaluation of artificial intelligence’s (AI) present application in glaucoma therapy, this review will concentrate on diagnosis, treatment optimization, and result prediction. A systematic approach to methodology ensured that high-quality and pertinent papers on AI applications in glaucoma were included.

Three main databases—PubMed, Scopus, and Web of Science—were searched extensively for relevant material. The goal of the search method was to find articles up to January 2024. The following set of Medical Subject Headings (MeSH) terms and keywords was employed: “machine learning”, “deep learning”, “glaucoma”, “diagnosis”, “artificial intelligence”, and “treatment”. To hone the search, the terms (AND/OR) were applied. The inclusion criteria were based on peer-reviewed original research articles, systematic reviews, and meta-analyses from January 1990 to September 2024. Glaucoma was diagnosed in human subjects. Research on AI-based methods used (machine learning, deep learning, neural networks) for glaucoma diagnosis, treating, or forecasting results was included. The exclusion criteria included papers that used animal models, studies that did not use AI or were solely concerned with using conventional diagnostic techniques, and certain article categories (letters, editorials, and case reports).

All articles found were imported into EndNote for de-duplication following the first search. The abstracts and titles were vetted for appropriateness by two separate reviewers using the inclusion criteria. Discussions with a third reviewer helped to settle disagreements. We gathered full-text publications of possibly pertinent studies and evaluated their eligibility. Articles that satisfied all requirements and offered adequate information on AI applications in glaucoma management were included in the final collection. The main goals of the study were how well AI models performed in glaucoma diagnosis, illness progression prediction, and treatment strategy optimization. Difficulties with model generalizability, interpretability, and clinical integration were among the secondary outcomes.

Despite the extensive availability of scientific studies, our research revealed a limited number of studies with substantial scientific evidence, with only 12 systematic reviews and meta-analyses (Table 1 and Table 2).

## 3. Machine Learning Models

Deep learning (DL) and machine learning (ML) are essential to AI-guided ophthalmic treatment transformation. ML algorithms, such as Support Vector Machine (SVM) and Random Forest (RF), are used to classify and interpret clinical data and imaging results, aiding in detecting disease patterns and anomalies. SVM is used for classification tasks to identify a boundary separating different data classes [25]. RF creates multiple decision trees and combines their outputs to improve prediction accuracy and robustness. DL, a more sophisticated subset of ML, leverages deep artificial neural networks to process large and multidimensional data. Convolutional Neural Networks (CNNs) excel in processing retinal and OCT images by automatically learning hierarchical features from raw pixel data through layers that detect patterns such as edges and textures. Other DL architectures, like Recurrent Neural Networks (RNNs) and Long Short-Term Memory (LSTM) networks, are well-suited for processing sequential and temporal data [26]. RNNs use their ability to maintain information over time to handle tasks like predicting sequences or analyzing time-series data. LSTM networks, a specialized type of RNN, enhance this capability by managing long-term dependencies in sequential data, making them ideal for complex temporal tasks. Meanwhile, generative adversarial networks (GANs) are employed to generate augmented datasets [7]. GANs consist of two neural networks (a generator and a discriminator) that work together to create and evaluate synthetic images, which helps generate realistic data to increase the existing datasets [27].

AI applications extend beyond mere image analysis; they include automatic image segmentation and enhancement, extracting detailed information that may be challenging to discern through traditional methods.

ML has emerged as a transformative tool in the medical field, enabling significant advancements in diagnostics, treatment planning, and patient management [27,28,29]. Its ability to analyze complex datasets and recognize patterns has led to enhanced predictive models that can improve clinical decision-making and outcomes. In ophthalmology, ML algorithms have been particularly impactful in detecting and classifying ocular diseases such as diabetic retinopathy, glaucoma, and age-related macular degeneration. These models, often based on deep learning architectures, have demonstrated accuracy comparable to or exceeding that of human experts, offering the potential for widespread deployment in screening programs and routine clinical practice [30].

For example, in glaucoma management, AI aids in interpreting intricate structural and functional data of the eye, optimizing diagnostic accuracy and treatment planning. Despite these advancements, challenges remain, such as ensuring high-quality data, managing biases, and improving model generalizability and interpretability.

ML and DL methods are revolutionizing glaucoma diagnosis and monitoring by providing cutting-edge instruments that can greatly increase the precision and effectiveness of clinical evaluations [31].

## 4. Results

### 4.1. Artificial Intelligence (AI) in Glaucoma

Our comprehensive search turned up about 400 articles about the use of artificial intelligence (AI) in the treatment of glaucoma. Based on the inclusion criteria, 60 papers were chosen for in-depth analysis after duplicates were eliminated and relevance was checked. The analysis focused on AI techniques in glaucoma diagnosis, therapy optimization, and outcome prediction. The papers under consideration examined a range of AI methods, with a particular emphasis on deep learning (DL) and machine learning (ML) models. Convolutional neural networks (CNNs) were the most widely used models for image analysis, particularly for fundus imaging and optical coherence tomography (OCT). The models exhibited encouraging precision in discerning glaucomatous damage, frequently matching the diagnostic precision of seasoned medical professionals. Nonetheless, there was a noticeable disparity in performance between the investigations, which was mostly related to variations in imaging technologies, population variety, and data quality.

Of the 60 papers, 35 mostly addressed the use of AI in glaucoma diagnosis. Analysis of structural alterations in the optic nerve head and retinal nerve fiber layer was commonly done using CNN-based models. Across a range of imaging datasets, these models demonstrated high sensitivity (from 83% to 96%) and specificity (from 80% to 92%). Most of these diagnostic investigations highlighted how AI could help with large-scale glaucoma screening programs, especially in impoverished areas where access to ophthalmologists is limited. Nevertheless, a few difficulties were identified as major constraints, including dataset biases and variations in image quality. According to a number of studies, in order to increase AI models’ generalizability across various populations, they must be trained on a variety of datasets.

AI was mostly employed to evaluate illness development and to optimize treatment options based on patient-specific data in the 25 studies that were primarily concerned with therapy and outcome prediction. Measurements of intraocular pressure (IOP), outcomes of visual field tests, and OCT scans were all part of the longitudinal data that machine learning models like Random Forest and Support Vector Machine (SVM) were applied to. These models demonstrated the ability to identify patients who were more likely to experience a rapid course of their condition, enabling earlier and more intensive management. Concerns were raised by several researchers on the shortcomings of the AI models used today to treat glaucoma. The absence of sizable, varied, and thoroughly annotated datasets remained a persistent problem, limiting the applicability of AI models to various patient populations and imaging technologies. Moreover, before many models can be safely incorporated into standard practice, they still need to be prospectively validated in actual clinical settings. It has also been common to debate ethical concerns about algorithmic transparency and data privacy, underscoring the necessity of strong regulatory frameworks prior to the widespread use of AI in clinical care.

Artificial intelligence (AI) has great promise for enhancing glaucoma care through better diagnosis, treatment, and outcome prediction. Even while the majority of AI models showed encouraging predictive and diagnostic accuracy, there are still many obstacles to overcome, especially when it comes to diverse data, validating models, and integrating AI into clinical workflows. In order to confirm the therapeutic efficacy of AI technologies in glaucoma therapy, future research should concentrate on resolving these constraints, especially by utilizing larger and more representative data. The current literature reports important trends, knowledge gaps, and future prospects for glaucoma research by combining the results of the included papers. These discoveries will play a pivotal role in steering the creation of resilient, broadly applicable, and therapeutically useful artificial intelligence systems.

The advent of AI is considered a medical revolution, including in ophthalmology [32]. Glaucoma is a leading cause of irreversible blindness worldwide, characterized by the progressive loss of retinal ganglion cells and corresponding visual field deficits. Early detection and consistent monitoring are crucial to prevent severe vision loss, yet the asymptomatic nature of early glaucoma makes it challenging to diagnose in its initial stages [33,34,35]. The first example of AI application in glaucoma dates to 1994, when Goldbaum et al. evaluated the reliability of trained neural networks in distinguishing between normal visual fields and those of glaucomatous eyes [36]. Later, in 1996, a study published in the American Journal of Ophthalmology by Brigatti et al. used various neural network algorithms to differentiate between normal and glaucomatous eyes using both structural data (cup/disk ratio, rim area, cup volume, and nerve fiber layer height) and functional data (visual field loss) [35]. From the 2000s onward, the application of increasingly sophisticated levels of AI to glaucoma has been on the rise, yielding progressively encouraging results and the development of numerous databases for training.

### 4.2. AI for Screening and Detection of Glaucoma

There is an increasing disparity between the demand for eye care and the availability of specialists. A 2010 international survey highlighted that, in developed nations, the population is rapidly aging, and there is a significant consequent shortage of well-trained ophthalmologists [37,38]. Integrating artificial intelligence into medical practice has shown the potential to enhance diagnostic capabilities, offering time-efficient and effective solutions to mitigate this gap. AI has shown important results in image-based diagnostics in ophthalmology, such as in the diagnosis of diabetic retinopathy [28,29]. AI has been widely applied in glaucoma diagnostics to achieve more accurate image segmentation/classification, enhance image quality, and support image interpretation [39,40]. In 2021, a meta-analysis by Buisson et al. demonstrated that even in a pessimistic model comparing the worst deep learning algorithms with the best ophthalmologists, the performance of deep learning in assessing the optic nerve head in fundus images is comparable to that of an ophthalmologist. This study included a total of 1.392 eyes from ophthalmic centers or datasets [41,42,43,44,45], with training datasets sourced from publicly available online retinal fundus image databases for glaucoma analysis and research (ORIGA), EyePacs, Inoveon, the Age-Related Eye Disease Study, and the United Kingdom Biobank [46,47,48]. This finding is consistent with the literature, and recent studies increasingly show that AI may outperform humans in evaluating optic nerve parameters in fundus examinations [49,50,51], partly due to the meticulous selection of high-quality images [52].

The performance of AI in evaluating the optic nerve from fundus images may be affected by several limitations, including the presence of optic nerve comorbidities (e.g., myopia), ethnic diversity in the sample, variations in image quality, differences between cropped and non-cropped optic disc images, and inconsistencies in comparisons between AI and human grading versus OCT or other objective data [53,54]. Regarding the latter point, some studies have shown that deep learning models trained with human grading make the same errors as a specialist, suggesting that comparisons with OCT data might be more effective [55,56].

On the other hand, the study by Phene et al. demonstrated that algorithms using more objective data achieved a worse AUC (receiver operating characteristic curve) compared to other groups [57].

A spectral domain (SD-OCT) is a crucial tool for the early diagnosis of glaucoma (before any visual field damage occurs) [43]. Integrating SD-OCT with AI would be extremely useful, as it is a widely available, not time-consuming, and highly repeatable examination [57,58]. The reliability of this diagnostic examination is closely dependent on the segmentation of the circumpapillary retinal layers. AI is currently being examined to better integrate OCT data into clinical practice and achieve the most accurate segmentation and analysis of OCT images.

According to the literature, the most recent innovations in SD-OCT image segmentation were proposed in 2023 by Gende M. et al. and in 2024 by Song Y et al. [59,60]. Gende et al. introduced the use of multiple view-specific modules to segment each scan pattern (circumpapillary circle scans, macular cube scans, and optic disc (OD) radial scans). This approach achieved better results for the cube and circle scan patterns compared to those obtained with state-of-the-art segmentation modules. Song et al. aimed to develop a segmentation method that is more efficient and less time-consuming. They studied a lightweight deep learning architecture for the simultaneous segmentation of the OC and OD, employing fuzzy learning and a multi-layer perceptron. This approach simplified learning complexity, improved image analysis accuracy, and demonstrated superiority over state-of-the-art methods. DL algorithms may outperform both traditional automated segmentation parameters and conventional machine learning classifiers (MLCs) in diagnosing glaucoma using OCT data. The authors developed a deep learning algorithm using OCT macular data from an 8 × 8 grid [61]. They employed transfer learning, first training the model on a pre-training dataset of 4.316 OCT images and then fine-tuning it with a smaller dataset of 178 images. The resulting DL model achieved an AUC of 0.937, with 83.3% sensitivity at 80% specificity, significantly surpassing the performance of both SVM (AUC = 0.82, sensitivity—39.5%, specificity—80%) and RF models (AUC = 0.674, sensitivity—35.1%, specificity—80%). In a prior 2017 study, Asaoka et al. had already developed an RF model incorporating circumpapillary retinal nerve fiber layer (RNFL) thickness data, achieving a comparable AUC of 0.93 [62]. A key finding is that their DL model maintained high diagnostic accuracy even when trained and tested on images from different OCT scanners, highlighting its robustness across various imaging devices. Segmentation-free approaches have also been proposed. For example, Thompson et al. demonstrated that using a DL algorithm trained on SDOCT circle B-scans without segmentation lines can effectively distinguish glaucomatous optic discs from healthy ones. In this case, the AUC for detecting glaucoma was significantly higher compared to global RNFL or sectoral RNFL obtained through automated segmentation (0.96 vs. 0.87; difference = 0.08 (95% CI: 0.04, 0.12), *p* < 0.001). Additionally, the performance was even better in discriminating cases of pre-perimetric or early glaucoma [63]. In 2019, Maetschke et al. developed a DL algorithm using unsegmented OCT volumes of the optic nerve head, achieving an AUC of 0.94, which significantly outperformed various MLCs (SVM 0.88; RF 0.86; extra trees 0.86; Naïve Bayes 0.86; LR 0.89; gradient boosting 0.82). It demonstrates that DL algorithms are superior to MLCs in detecting glaucoma on OCT [54]. The limitations of current studies on OCT and AI mainly involve segmentation errors, the limited ethnic diversity in the datasets, and the exclusion of cases of secondary glaucoma or angle-closure glaucoma [64,65,66,67]. Addressing these issues would improve the generalizability of the findings.

### 4.3. AI and Assessment of SAP (Standard Automated Perimetry)

Standard Automated Perimetry (SAP) is a cornerstone in the functional assessment of glaucoma, providing critical information about visual field defects that are indicative of disease progression. However, the interpretation of SAP results can be challenging due to variability in patient responses, the subtle nature of early defects, and the complexity of the data generated. AI has been increasingly applied to enhance the accuracy and reliability of SAP assessments by automating the analysis of visual field data and identifying patterns that may elude traditional statistical methods. AI models, particularly those based on ML and DL, have demonstrated significant potential in improving the interpretation of SAP data [68,69]. These models can be trained to recognize complex visual field patterns associated with glaucomatous damage, including both localized and diffuse loss, which may not be immediately apparent to human observers. By leveraging large datasets of SAP results, AI can provide a more consistent and objective assessment of visual field progression, reducing the subjectivity inherent in manual interpretation [70]. Moreover, AI-driven approaches have been developed to predict future visual field deterioration by analyzing trends in SAP data over time. This predictive capability is especially valuable for personalized glaucoma management, allowing clinicians to tailor treatment strategies based on the likelihood of disease progression. Additionally, AI can assist in identifying artifacts and unreliable test results, thereby improving the overall quality and reliability of SAP data. Despite these advancements, challenges remain in integrating AI into routine clinical practice. These include ensuring the generalizability of AI models across diverse populations, maintaining transparency in AI-driven decision-making, and addressing potential biases in training data. Furthermore, developing user-friendly interfaces for AI tools is essential to facilitate their adoption by clinicians without specialized technical expertise. AI offers promising advancements in assessing SAP, enhancing both the accuracy of glaucoma diagnosis and the monitoring of disease progression [71].

### 4.4. AI and OCT-A “New Application”

Angio-OCT (OCT-A) is a noninvasive imaging modality that provides a detailed visualization of retinal and choroidal blood vessels, offering critical insights into vascular conditions such as diabetic retinopathy, age-related macular degeneration, and glaucoma. However, the complexity and volume of data generated by Angio-OCT pose significant challenges for manual interpretation. AI, particularly through DL algorithms, offers a powerful solution by automating the analysis of these high-dimensional datasets, facilitating more accurate and efficient diagnoses [72].

AI algorithms applied to Angio-OCT can detect and quantify microvascular changes with a level of precision that surpasses conventional methods. For example, deep learning models have been trained to automatically segment retinal layers, identify regions of non-perfusion, and detect neovascularization with high sensitivity and specificity. These capabilities are crucial for the early diagnosis and monitoring of retinal diseases, where punctual intervention can prevent significant vision loss [73]. Moreover, AI can integrate Angio-OCT data with other imaging modalities and clinical information, providing a comprehensive ocular health assessment and supporting personalized treatment strategies. One of the most promising applications of AI in Angio-OCT is its potential for use in population-based screening programs. By automating the detection of pathological features, AI-driven Angio-OCT can identify individuals at risk of sight-threatening diseases in large-scale screenings, thereby improving access to care and reducing the burden on healthcare systems. Additionally, the ability of AI to learn and adapt from new data continually enhances its diagnostic performance, making it a dynamic tool in clinical practice [74]. Challenges such as the need for large, annotated datasets, model generalizability, and the integration of AI tools into clinical workflows must be addressed. Ethical considerations, including data privacy and the interpretability of AI decisions, are also critical to ensuring the safe and effective use of these technologies.

The combination of AI and angio-OCT heralds a new era in retinal imaging, offering unprecedented opportunities for the early diagnosis, monitoring, and management of retinal diseases. Continued research and collaboration across disciplines will be key to unlocking this technology’s full potential in clinical practice [75].

### 4.5. AI and Anterior Segment Evaluation (AS-OCT)

Artificial intelligence is transforming anterior segment evaluation via anterior segment optical coherence tomography (AS-OCT). AI algorithms, especially DL models, excel in analyzing AS-OCT images by automatically segmenting structures such as the cornea, iris, and lens. These algorithms can accurately measure parameters like corneal thickness, anterior chamber depth, and angle opening distance, which are critical in diagnosing conditions like keratoconus, angle-closure glaucoma, and post-surgical assessments [76,77].

AI enhances diagnostic precision by identifying subtle anatomical changes and reducing inter-observer variability. It facilitates the early detection of pathologies and enables personalized treatment planning. The primary challenge lies in the need for extensive, diverse datasets to train these models effectively, ensuring they generalize well across different populations and AS-OCT devices [78,79,80].

### 4.6. AI: Support in Treatment and Outcome Prediction

Given the progressive and often asymptomatic nature of glaucoma, personalized treatment plans are essential for preserving vision and preventing irreversible damage. AI-driven models, particularly those based on ML and DL, have demonstrated substantial potential in optimizing glaucoma management by analyzing large datasets, including clinical records, imaging data, and patient demographics [34]. The integration of AI in the management of glaucoma has opened new avenues for enhancing treatment strategies and predicting patient outcomes with unprecedented accuracy. Central to this advancement is the utilization of ML algorithms, particularly DL techniques, which have demonstrated remarkable efficacy in interpreting complex ocular data. For example, CNNs have been employed to analyze high-resolution images from OCT and fundus photography, enabling the early detection of glaucomatous changes in the RNFL and optic nerve head (ONH). These algorithms can detect subtle structural alterations that may not be visible to the human examiner, thereby facilitating earlier and more accurate diagnoses. One of the key applications of AI in glaucoma treatment is in the stratification of patients based on their risk of disease progression.

AI models can identify patients at higher risk of rapid progression by analyzing longitudinal data from visual fields, OCT scans, and intraocular pressure (IOP) measurements. This enables us to tailor treatment plans more effectively by intensifying therapy or scheduling more frequent follow-ups for high-risk individuals. In addition to diagnostic support, AI has shown significant promise in predictive modeling, a critical aspect of glaucoma management. By leveraging large datasets that include longitudinal patient data, such as IOP readings, visual field test results, and genetic factors, AI models can predict the progression of the disease with a high degree of accuracy. Moreover, AI can predict the response to different treatment modalities, such as medications, laser therapy, or surgical interventions, by correlating past patient responses with various treatment outcomes. This predictive capability allows for a more personalized approach, potentially reducing the trial-and-error period often associated with glaucoma management. Additionally, AI tools are continuously being developed to monitor treatment efficacy [35,69,81]. For example, AI algorithms can automatically analyze visual field test results and OCT scans over time to detect subtle changes that may indicate either disease progression or improvement. This continuous monitoring is crucial for adjusting treatment plans promptly, thereby minimizing the risk of vision loss. Furthermore, AI can help predict long-term outcomes, such as the likelihood of maintaining functional vision, based on initial presentation and early treatment response, thus aiding in patient counseling and expectation management [82]. This predictive capability is valuable, allowing for timely, more aggressive interventions. For instance, AI-based models can simulate various treatment scenarios, providing insights into the potential outcomes of different therapeutic approaches, which helps optimize treatment plans tailored to individual patients.

Moreover, AI systems have the potential to revolutionize the way clinicians manage glaucoma by incorporating real-time data analysis and decision support into clinical practice. These systems can be used to continuously monitor patient responses to treatments, adjusting recommendations based on the latest data and providing personalized treatment regimens that maximize efficacy while minimizing side effects. This dynamic and data-driven approach to glaucoma management not only enhances the precision of care, but also promises to significantly reduce the burden of blindness associated with this condition by enabling earlier, more effective interventions [28]. ML models have been tested for their capacity to predict real-life trabeculectomy results using easily accessible preoperative patient demographic, ocular, and systemic health data [83]. To predict the overall failure and results of medical and surgical glaucoma therapy, several ML classifiers were trained, including decision trees, Random Forests, XGBooste 2.1.0 (Xtreme Gradient Boosting), penalized logistic regression, multi-layer perceptrons, k-nearest neighbors, Gaussian naive bayes, and linear discriminant analysis.

Standard classification metrics were used to assess each model on the provided dataset. These metrics included accuracy, precision (positive predictive value), negative predictive value, recall (sensitivity), specificity, accuracy, area under the receiver operating characteristic curve (AUROC), area under the precision–recall curve, and F1-score (the harmonic mean of precision and recall). According to the authors, the most effective algorithms were neural network and random forest models, which demonstrated significant predictive power given their complexity [84].

## 5. Discussion

Integrating AI into clinical practice is not without challenges. Ensuring the accuracy and generalizability of AI models across diverse patient populations and clinical settings is essential. Additionally, the interpretability of AI-generated predictions remains a concern, as clinicians need to understand and trust the reasoning behind AI recommendations. Addressing these challenges requires ongoing research, large-scale validation studies, and the development of user-friendly interfaces that seamlessly integrate AI tools into routine clinical workflows. AI has the potential to significantly enhance the treatment and outcome prediction of glaucoma, leading to more personalized and effective care. As AI technology evolves, it is poised to become integral to glaucoma management, ultimately improving patient outcomes and quality of life [81].

Despite the advancements in AI technology, clinicians remain indispensable in the AI-driven management of glaucoma. While AI can process complex data and identify patterns beyond human capacity, clinical expertise is required to interpret these findings in the context of individual patient care. Clinicians are responsible for integrating AI-derived insights with real-world considerations, such as patient-specific risk factors, preferences, and comorbidities, ensuring that AI tools are used to support, rather than replace, clinical decision-making. For instance, AI may identify subtle changes in ocular imaging, but it is the clinician who must determine the appropriate course of action, such as adjusting treatment or scheduling further follow-up. As AI continues to evolve, its integration into routine practice will depend heavily on its ability to complement clinical workflows, with clinicians maintaining oversight and accountability for patient outcomes.

AI in glaucoma primarily involves ML and DL algorithms that analyze large datasets, including OCT images, fundus photographs, and visual field tests. These algorithms can identify subtle changes in the retinal nerve fiber layer thickness, optic nerve head structure, and visual field patterns, which may not be readily apparent to human clinicians. By training on labeled datasets, AI systems can achieve diagnostic accuracy comparable to glaucoma specialists [61].

One of AI’s most significant contributions to glaucoma is its application in automated screening. For instance, DL models have been developed to analyze OCT images and detect glaucomatous damage with high sensitivity and specificity. These models are particularly valuable in community-based screening programs, where access to ophthalmic specialists may be limited. Furthermore, AI-driven tools are being used to predict the risk of glaucoma progression by analyzing longitudinal data from visual fields and OCT scans, enabling personalized treatment plans [85].

While AI has shown great potential in glaucoma diagnosis and treatment, AI’s incorporation into standard clinical practice is still in its infancy. Deep learning algorithms in particular have shown remarkable accuracy in detecting glaucomatous alterations using imaging modalities including fundus photos and optical coherence tomography (OCT). Before AI is extensively employed for clinical diagnosis and treatment, there are significant restrictions to take into account. It has been observed that the intricacy and subtleties of clinical diagnosis performed by ophthalmologists are not yet matched by AI technology, and there is still a sizable discrepancy between AI forecasts and actual clinical decision-making.

The need for more randomized controlled trials (RCTs) utilizing sizable, varied datasets that reflect a range of people, ethnicities, and equipment brands is one of the main obstacles. It is improbable that AI models developed on small or homogeneous datasets will perform well in larger populations. Future research must concentrate on collecting high-quality data from a wide range of demographic groups and imaging technologies in order for AI to be successfully integrated into the treatment of glaucoma. By doing this, biases will be lessened, and it will be guaranteed that AI technologies are trustworthy and fair in various clinical contexts. Furthermore, in order to evaluate AI tools’ efficacy in actual clinical settings, randomized controlled trials (RCTs) must validate them. Currently available AI models are frequently tested in controlled contexts or retrospectively, and these may not accurately capture the intricacies present in routine clinical practice.

Prospective randomized controlled trials (RCTs) will offer more robust proof of the usefulness of AI tools by contrasting diagnoses made by AI with those made by skilled medical professionals, and establishing if AI can, in fact, enhance patient outcomes in the treatment of glaucoma. AI-based diagnoses will not completely replace clinical expertise; rather, they will serve as a supplement until these thorough studies are finished and the data are polished. This thorough assessment of AI’s shortcomings emphasizes the necessity of ongoing study and development prior to the full integration of AI in the diagnosis and management of glaucoma. AI in ophthalmology has a bright future ahead of it, but in order to achieve widespread clinical application, these obstacles must be overcome. This may be done by working together, validating AI models on a large scale, and continuously improving AI models [86].

Ongoing efforts to address this gap in evidence are reflected in the current landscape of clinical trials. According to the Cochrane Library, several trials are underway to assess the clinical utility of AI in glaucoma. These studies aim to evaluate AI algorithms’ diagnostic accuracy, their ability to predict disease progression, and their effectiveness in guiding treatment decisions [87]. However, the outcomes of these trials are awaited, and it is crucial that they are conducted with rigorous methodologies, including adequate sample sizes, diverse patient populations, and standardized outcome measures [88].

Moreover, future research should not only focus on the technical performance of AI models but also their real-world applicability, considering factors such as user acceptance, cost-effectiveness, and integration into existing healthcare infrastructures. Ethical considerations, including data privacy and the transparency of AI decision-making processes, must also be addressed to ensure AI technologies’ safe and equitable implementation. AI has the potential to revolutionize glaucoma care. Still, there is a pressing need for high-quality, randomized controlled trials to provide the necessary evidence for its widespread adoption in clinical practice. The results of ongoing and future trials will be pivotal in determining whether AI can fulfill its promise of improving glaucoma outcomes on a broad scale [89].

Furthermore, one of the most significant challenges in applying AI to glaucoma diagnosis and management is the quality and comprehensiveness of the datasets used to train AI models. The performance and reliability of AI algorithms are inherently dependent on the data they are trained on, and limitations in these datasets can lead to biases, reduced accuracy, and the poor generalizability of AI tools in real-world clinical settings [90].

A primary concern is the issue of data diversity. Many AI models for glaucoma have been trained on datasets that lack adequate representations of various demographic groups, such as different ethnicities, ages, and stages of glaucoma. This lack of diversity can result in AI models that perform well in specific populations but poorly in others, leading to inequitable care and potential harm. For example, glaucoma manifests differently across populations, and AI systems trained predominantly on data from one group may fail to diagnose or predict outcomes for individuals from other groups accurately. Addressing this requires the inclusion of diverse and representative datasets in the training process to ensure that AI models are robust and applicable to a wide range of patients. Another critical limitation is the size of the datasets. AI, particularly DL models, requires large volumes of data to achieve high levels of accuracy. However, the availability of such large datasets, especially with well-annotated, high-quality data, is often limited in the field of ophthalmology. Small or poorly annotated datasets can lead to overfitting, where the model performs well on the training data but fails to be generalizable to new, unseen data. This problem highlights the need for collaborations across institutions and even countries to aggregate large, diverse datasets that can better train AI models [91].

Moreover, the quality of data is also a significant concern. Datasets that contain noise, inconsistencies, or errors can degrade the performance of AI models. In the context of glaucoma, where small changes in visual field tests or imaging can have significant clinical implications, the quality of the input data is paramount. Ensuring high-quality data collection and curation processes and standardizing data across different imaging devices and clinical environments is essential for developing reliable AI tools.

Lastly, there is a need to address the limitations related to the historical data used in AI models. Many datasets may not capture recent advances in imaging technology or treatment protocols, which could result in AI models that are out of sync with current clinical practices. The continuous updating of AI models with new data is necessary to maintain their relevance and accuracy over time. Addressing the limitations of datasets is crucial when developing reliable and equitable AI models in glaucoma care. This requires ensuring data diversity, increasing dataset sizes, improving data quality, and keeping models updated with the latest clinical information. Overcoming these challenges will enhance the robustness and generalizability of AI, leading to better patient outcomes across diverse populations.

## 6. Conclusions

Despite these promising advancements, there are several challenges to the widespread adoption of AI in glaucoma. These include the need for large, diverse, high-quality datasets, model interpretability, and the integration of AI tools into clinical workflows. Moreover, ethical considerations such as patient privacy, data security, and the potential for algorithmic bias must be addressed to ensure the responsible use of AI in ophthalmology. In conclusion, AI has the potential to revolutionize the diagnosis and management of glaucoma by providing accurate, efficient, and scalable solutions.

Continued research and collaboration between clinicians, data scientists, and engineers are essential to overcome current limitations and fully realize the benefits of AI in glaucoma care.

## Figures and Tables

**Table 1:** Systematic reviews related to AI and glaucoma.

Title	Authors	Year	Journal	Purpose
Artificial Intelligence in Anterior Chamber Evaluation: A Systematic Review and Meta-analysis	Olyntho MAC Jr et al. [2]	2024	J Glaucoma	To compare the accuracy of deep learning algorithms (DLA) applied to anterior segment optical coherence tomography images (AS-OCT) against gonioscopy in detecting angle closure in patients with glaucoma.
Artificial intelligence for the detection of glaucoma with SD-OCT images: a systematic review and Meta-analysis	Shi NN et al. [3]	2024	Int J Ophthalmol	To quantify the performance of artificial intelligence (AI) in detecting glaucoma with spectral domain optical coherence tomography (SD-OCT) images
A systematic review of economic evaluation of artificial intelligence-based screening for eye diseases: From possibility to reality	Wu H et al. [4]	2024	Surv Ophthalmol	Quantitative analysis of health economics concerning AI
Clinical applications of anterior segment swept-source optical coherence tomography: A systematic review	Mirzayev I et al. [5]	2023	Photodiagnosis Photodyn Ther	Utilization of AS SS-OCT in various conditions, including glaucoma, ocular surface pathologies, iris tumors, refractive surgery, cataract surgery, scleral diseases, and AI
Anterior segment optical coherence tomography (AS-OCT) image analysis methods and applications: A systematic review	Garcia Marin YF et al. [6]	2022	Comput Biol Med	To provide an in-depth summary and to classify image analysis techniques found in the literature applied to AS-OCT images (including AI)
Performances of Machine Learning in Detecting Glaucoma Using Fundus and Retinal Optical Coherence Tomography Images: A Meta-Analysis	Wu JH et al. [7]	2022	Am J Ophthalmol	To evaluate the performance of machine learning (ML) in detecting glaucoma using fundus and retinal optical coherence tomography (OCT) images
Deep learning versus ophthalmologists for screening for glaucoma on fundus examination: A systematic review and meta-analysis	Buisson M et al. [8]	2021	Clin Exp Ophthalmol	To compare deep learning versus ophthalmologists in glaucoma diagnosis on fundus examinations
Accuracy of Using Generative Adversarial Networks for Glaucoma Detection: Systematic Review and Bibliometric Analysis	Saeed AQ et al. [9]	2021	J Med Internet Res	To illustrate deep adversarial learning as a potential diagnostic tool and the challenges involved in its implementation
Diagnostic accuracy of deep learning in medical imaging: a systematic review and meta-analysis	Aggarwal R et al. [10]	2021	NPJ Digit Med	To evaluate the diagnostic accuracy of using DL algorithms to identify pathology (like glaucoma) in medical imaging
Applications of deep learning in the detection of glaucoma: A systematic review	Mirzania D et al. [11]	2021	Eur J Ophthalmol	DL methods to detect glaucoma using color fundus photographs, optical coherence tomography (OCT), or standard automated perimetry (SAP)
Deep Learning for Accurate Diagnosis of Glaucomatous Optic Neuropathy Using Digital Fundus Image: A Meta-Analysis	Islam M et al. [12]	2020	Stud Health Technol Inform	To investigate the performance of deep learning algorithms for the detection of GON (glaucomatous optic neuropathy)
Current applications of machine learning in the screening and diagnosis of glaucoma: a systematic review and meta-analysis	Murtagh P et al. [13]	2020	Int J Ophthalmol	To compare the effectiveness of two well-described machine learning modalities, ocular coherence tomography (OCT) and fundal photography, in terms of diagnostic accuracy in the screening and diagnosis of glaucoma

**Table 2:** Literature review on deep learning/machine learning and glaucoma *.

Study	Purpose	AI Technique	Data Used	Key Findings	Publication
Wu et al. (2022) [7]	Evaluate AI in glaucoma detection.	Machine Learning	Fundus and OCT images	High diagnostic accuracy	Am J Ophthalmol
Ran et al. (2021) [14]	Review of DL models in glaucoma	Deep Learning	OCT images	Strengths and limitations of DL in diagnosis	Eye
Akter et al. (2022) [15]	Glaucoma diagnosis using deep learning	Deep Learning	Retinal images	Improved diagnostic performance	Scientific Reports
Zhang et al. (2022) [16]	Predict glaucoma progression	Deep Learning	Fundus photographs	AUROC 0.90 for incidence prediction	Ophthalmology
Muhammad et al. (2017) [17]	Classify glaucoma suspects	Hybrid Deep Learning	Wide-field OCT	Accurate suspect classification	Journal of Glaucoma
Andersson et al. (2013) [18]	Compare AI vs. clinicians in diagnosis	ANN **	Visual field data	Comparable accuracy with clinicians	Acta Ophthalmologica
Ran et al. (2022) [19]	DL for glaucoma using OCT	Deep Learning	OCT Images	High accuracy in detecting progression	Eye
Barella et al. (2013) [20]	Evaluate ML classifiers for glaucoma	Machine Learning	Retinal nerve fiber layer data	High diagnostic accuracy	J Ophthalmol
Yousefi et al. (2016) [21]	Detect glaucomatous progression	Gaussian Mixture Model	Visual fields	Effective in progression detection	Transl Vis Sci Technol
Zhang et al. (2021) [22]	DL for glaucoma risk prediction	Deep Learning	Fundus photographs	Effective risk stratification	Ophthalmology
Wagner et al. (2022) [23]	Updates on AI in glaucoma diagnosis	Systematic Review	Various AI models	Summarized recent advances	Mayo Clin Proc Innov Qual Outcomes
Seker et al. (2022) [24]	Address bias in AI healthcare data	Data Preprocessing	Various datasets	Recommendations for bias mitigation	Stud Health Technol Inform

* Each study evaluates different aspects of AI’s role in glaucoma ** Artificial neural network.

## Data Availability

Not applicable.
